# Immunotherapeutic strategy in the management of gastric cancer: molecular profiles, current practice, and ongoing trials

**DOI:** 10.1186/s43046-023-00192-1

**Published:** 2023-10-02

**Authors:** Mengxiao Lu, Yingjie Wu, Yixin Zhang, Yu Yu, ShengJie Wang, Xiaobao Su

**Affiliations:** 1 Department of Gastrointestinal Minimally Invasive Surgery, The Affiliated People’s Hospital of Ningbo University, Ningbo, China; 2 Ningbo University, Ningbo, China

**Keywords:** Gastric cancer, Immunotherapy, Immune checkpoint inhibitors, PD-L1

## Abstract

Gastric cancer (GC) is the one of the most commonly solid cancer worldwide. Although under the aggressive treatment, the poor clinical outcomes of patients with GCs have not been improved. Current studies emphasized that targeting therapies or immune response-based therapeutic strategy may be a potential approach to improve the clinical outcomes. Moreover, accumulative evidence has reported the increasing expression of PD-L1 expression in GC cells and highlighted its role in the tumor progression. Currently, great development has been established in the immune checkpoint inhibitors (ICIs) and further changed the clinical practice of GC treatment and prognosis. In addition, the combination therapies with targeting therapy or traditional therapies are expected to push the development of immunotherapies. In our present review, we predominantly focus on the biomarkers and molecular profiles for immunotherapies in GCs and highlight the role and administration of ICIs-based immunotherapeutic strategies against the GCs.

## Introduction

Gastric cancer (GC) is one of the most commonly solid cancer worldwide [1]. Because of the late diagnosis of GCs, the patients with GCs always present poor clinical outcomes. Accumulative evidence has revealed that the 5-year survival rate of patients with GCs is only 20–30% [2, 3]. Currently, the immunotherapy has been incorporated in the clinical management and widely explored as a potential therapeutic strategy [3]. The existing immunotherapies can be classified as the active and passive approaches. The active immunotherapies utilize the immune responses in patient per se to destroy the cancer cells, while the passive immunotherapies depend on the exogenous agent, like targeting antibody to destroy cancer cells. Immunotherapies against the tumor have brought hope to amount of patients, especially in GCs [4]. With the breakthrough development of immunotherapies both in experimental study and clinical trials, a variety of immunotherapies are available for patients with GCs, and novel approaches are under clinical investigation. Among various immunotherapies, administration with immune checkpoint inhibitors (ICIs) has been served as the standard of care management recently. In our present review, we predominantly focus on the biomarkers and molecular profiles for immunotherapies in GCs and highlight the role and administration of ICIs-based immunotherapeutic strategies against the GCs.

## Biomarkers and molecular profiles for immunotherapies in GC

Accumulative evidence has revealed that tumor cells can escape the immune responses and surveillance through various mechanisms. Among them, immunoinhibitory checkpoints (programmed cell death protein-1 (PD-1)/programmed death ligand-1 (PD-L1) and cytotoxic T-lymphocyte-associated antigen 4 (CTLA-4)) mediated co-inhibitory pathways presented a critical role in immunosuppression [5]. PD-1/PD-L1 and CTLA-4 have been served as target, and the immune checkpoint inhibitors (ICIs) have been established to reactivate immune responses within the tumor microenvironment in a variety of cancers. During the latest years, immunotherapeutic strategies have been widely accepted in clinical practice as the part of first-line treatment against advanced gastric cancer (GC) [6]. According to previous studies, it has been reported that different immunotherapeutic strategies present diverse efficacy in patients with GC, which makes it a huge challenge to evaluate the clinical benefits of ICIs. Therefore, it is necessary to determine biomarkers and molecular profiles to predict the efficacy of immunotherapies against GCs. So far, there are several potential biomarkers to assess the therapeutic responses after ICIs therapy, including PD-L1 levels, tumor mutational burden (TMB), microsatellite instability-high (MSI-H), Epstein-Barr virus (EBV) infection, gut microbiota, and HER2 overexpression [7–9].

### Evaluation of PD-1 expression levels

There are three criteria of PD-L1 level in GCs. Of note, PD-L1 combined positive score (CPS) is a better assessing approach in patients of GC and has been served as a predictive marker of the efficacy of ICIs for advanced gastric cancer and a stratification factor in clinical practice. According to the CheckMate-032 study, it has been reported that CPS is a better evaluation criteria than tumor proportion score (TPS) in assessing PD-L1 expression levels [10]. Moreover, in the KEYNOTE-061 study, it has been revealed that patients with *CPS* ≥ 10 benefit most from the pembrolizumab [11]. Due to the results of CP-MGAH22–05 study, dual-positive subgroup (HER2 and PD-L1) presented longer PFS and OS, which revealed the efficacy of combination of margetuximab and pembrolizumab was related to the expression of PD-L1 [12]. In the contrary, according to the ATTRACTION-2 study, the benefits of nivolumab on overall survival (OS) were not associated with the PD-L1 expression levels [13]. On the contrary, some studies on various solid tumors assessed the PD-L1 levels by TPS rather than CPS. CPS is generally assessed through the biopsy tissue [14]. Therefore, CPS is not a perfect but a useful biomarker for advanced GCs patients with ICIs treatment.

### Tumor mutational burden (TMB)

TMB is a useful biomarker of ICI response in a wide range of solid tumors, including bladder cancer, melanoma, and glioma. TMB refers to the quantification of the number of somatic mutations in coding region of the genome. According to both of the KEYNOTE-061 and KEYNOTE-158 trial, TMB-H patients obtained higher ORR, longer OS, and significant clinical benefit comparing to the non-TMB-H tumors [11, 15, 16]. Due to the positive correlation of TMB and clinical ICIs responses, TMB is potentially served as the immunotherapy-related biomarker [17]. A previous study reported that patients with advanced TMB-H GCs treated with systemic therapy in clinical practice presented better outcomes than TMB-L patients [18]. Therefore, pembrolizumab was granted approval for TMB-H patients. However, further investigation with large cohort is also required to determine the role TMB-H for patients with GCs.

### Microsatellite instability-high (MSI-H)

MSI-H is caused by the MMR gene defect and is also a predictor for ICIs responses. For gastric cancer, MSI-H status is a favorable prognostic factor, a positive predictor for ICIs response, and a negative predictor of cytotoxic chemotherapy responses. Accumulative evidence revealed the relationship of the ICIs efficacy in patients with MSI-H tumors. According to the phase III KEYNOTE-062 trial, classified through the PD-L1 CPS, combination of pembrolizumab and chemotherapy presented no significant more benefits on OS in patients (*CPS* ≥ 10 or *CPS* ≥ 1) than chemotherapy alone. However, patients with MSI-H tumors obtained more benefits from combination of pembrolizumab and chemotherapy treatment than chemotherapy alone [19]. Notably, MSI-H GCs also present TMB-H status, indicating the potentially promoted immune cell or ICIs responses.

### Epstein–Barr virus (EBV) infection status

EBV is the predominant virulence factor for nasopharyngeal carcinoma. Current evidence also revealed that EBV infection can induce the progression of EBV-associated GC (EBVaGC) [20]. Latent EBV proteins can downregulate the expression of E-cadherin, which also is the important step in the progression of the loss of cell-to-cell adhesion and the carcinogenesis of EBVaGC [21]. Moreover, it has also been demonstrated that the EBV miRNA BART11 can reduce the expression of forkhead box protein P1 (FOXP1), which activates the epithelial-mesenchymal transition (EMT) of GCs and further accelerates cancer invasion and metastasis [22]. According to the previous study, there is a positive association between EBV status and CD8-positive T-cell infiltration and PD-L1 expression in EBVaGC, suggesting its great sensitivity to ICIs. Accumulative evidence has revealed that EBV status was positively associated with the expression of PD-L1 [23, 24]. Therefore, it has been reported that ICIs were successful against EBVaGC and MSI GCs [25]. A phase II study of pembrolizumab demonstrated that EBVaGC was more susceptible to the administration ICIs [26].

### Overexpression of human epidermal growth factor receptor 2 (HER2)

HER2 is a receptor tyrosine kinase proto-oncogene and has been attached great attention in GCs. The overexpression of HER2 can be observed in approximately 17.9% of GCs [27]. Moreover, the overexpression of HER2 is also associated with the poor clinical prognosis and increased recurrence in GCs [28]. Previous study revealed that targeting HER2 with the monoclonal antibody trastuzumab combined with the chemotherapy can prolong the survival of patients with HER2-positive GCs [1]. However, only limited benefits can be obtained in overall survival [29].

Overexpression of HER2 also is associated with the increasing expression of PD-L1. According to a previous study, 85% of HER2-positive GCs were featured by the overexpression of PD-L1 [30].

Based on an experimental study, downregulation of HER2 in PD-L1/HER2-positive GC organoids leads to a decrease in PD-L1 expression [31]. Above results indicated that combination of PD-L1 targeting therapy and anti-HER2 therapy may present a potential and positive effects in patients with HER2-positive GCs.

### Immune microenvironment

The tumor microenvironment (TME) presents a critical role in immune escape and resistance against cancer therapies, leading to the progression of malignancy. During the therapeutic process, TME contributes to the oncogenesis and therapeutic efficacy. The tumor-infiltrating immune cells are the predominant components within the TMW and present a wide range of functions. Within the TME of GCs, the most predominant immune cells are tumor-associated macrophages (TAMs) and tumor infiltrating lymphocytes (TILs). In addition, it has been reported that HER2 also mediate the alteration of TME, which further affect the tumor progression and clinical prognosis.

The TAMs derived from the peripheral blood then infiltrate the tumor tissues and release a variety of chemokine to affect the tumor growth, invasion, and metastasis. Several properties of TAMs have been served as predictors in GC, including TAM density or TAM polarization. In addition, a variety of TAM-derived factors are also considered as the biomarker to predict the clinical outcomes, involving Tim-3 or CCL5. Of note, TAM can induce the immune tolerance by blocking the anti-tumor function of cytotoxic T cell. It has also been reported that TAMs present a critical role in angiogenesis within TME. TILs are another key immune component in GCs, which are comprised of B cells, T cells, and nature killer (NK) cells. It has been reported that TILs are the predictor of poor clinical outcomes or tumor recurrence in GCs. Of note, increasing infiltration of CD8-positive lymphocytes is associated with prolonged OS in GCs, while high density of Th22 and Th17 cells related to a decreased OS. Immune evasion is a critical step during the progression of GCs, which is mediated by PD-L1. The tumor-localized PD-L1 can bind to the PD-1 on lymphocytes, which inhibit the anti-tumor immune responses. Therefore, PD-L1 may be useful targets in the management of GCs.

Dendritic cells (DCs) are another critical component within immune microenvironment in GC, which is responsible for presenting antigen towards immune cells and mediate further immune responses. It has been reported that increasing DC infiltration was associated with the increased 5-year survival in GC [32]. According to a previous clinical trial, administration with tumor-associated antigens, including HER2 peptide, can activate DCs, which can be autologous transplanted into patients and induce T-cell response against the antigen [33]. Currently, the GCs have been classified into four subtypes through the Cancer Genome Atlas (TCGA), including EBV, MSI, genomically stable (GS), and CIN. There is intense infiltration of lymphocytes which can be observed in the EBV and MSI-H subtypes. The GS subtype presents more CD4-positive T cells, macrophages, and B cells, which is more suitable for immunotherapies. Furthermore, the CIN subtype presents T-cell depletion and more infiltration of TAMs, considered as the “cold tumors.”

## Immunotherapy in the clinical management against GC

### ICIs: PD-1/PD-L1 inhibitors and anti-CTLA4 antibodies

PD-1 is a negative costimulatory immune molecule localized in the surface of various immune cells. Its corresponding receptor PD-L1 is localized on antigen-presenting cells (APCs) and tumor cells. The binding between PD-1 and PD-L1 activates immunosuppressive signal pathways and mediate immune escape. Therefore, inhibiting this interaction can promote the immunotherapeutic responses. According to the phase I study KEYNOTE-012, it has been reported that pembrolizumab presented a potential anti-tumoral function in advanced GCs [34]. In the following study, KEYNOTE-059 cohort 1 revealed that pembrolizumab monotherapy presented a significant efficacy. Therefore, the FDA approved pembrolizumab as a third-line treatment for patients with advanced or metastatic GC (PD-L1 *CPS* ≥ 1). However, the KEYNOTE-061 study reported that pembrolizumab failed to present advantage than chemotherapies in patients with PD-L1-positive GCs [11]. Moreover, nivolumab has also been conducted as the PD-L1 inhibitor in ATTRACTION-2 study. The results indicated that great benefits on OS can be observed by using nivolumab [13]. An exploratory analysis on the avelumab treatment revealed that prolonged OS in patients with PD-L1 *CPS* ≥ 1. However, in the JAVELIN Gastric 100 study, avelumab administration after the first-line chemotherapies in advanced GC fails to improve the OS in patients with PD-L1 *TPS* ≥ 1% [35]. Taken together, although the PD-1/PD-L1 inhibitors present potential clinical efficacy against GCs, the benefits of monotherapy are still limited. Therefore, the combination of ICIs and chemotherapies may present more clinical significance. So far, there are four pivotal phase III trials which have been published to assess the efficacy of ICIs for advanced GCs.

**Fig. 1:**
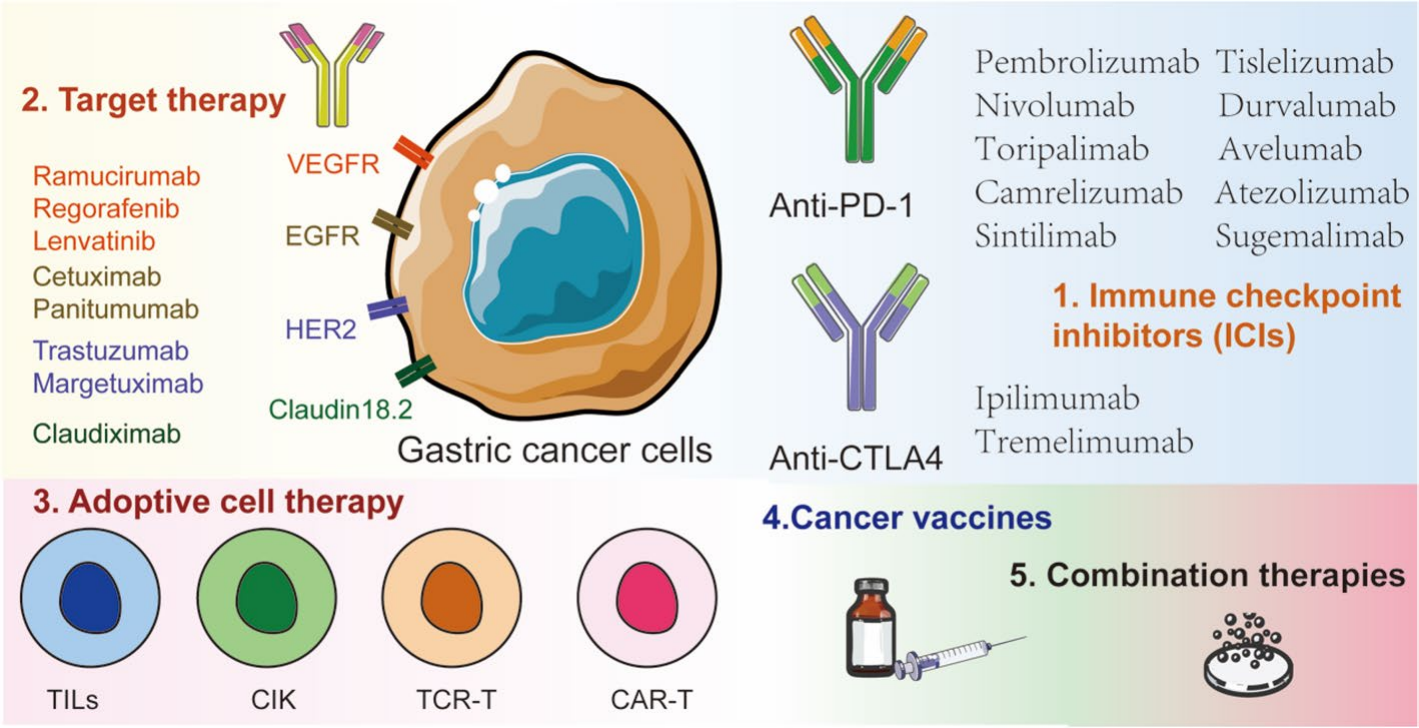
Different immunotherapeutic strategies against gastric cancer. Immune checkpoint inhibitor (ICIs), adoptive cell therapy (ACT), target therapy, cancer vaccine, and combination therapy are the predominant types of immunotherapies against GCs

CTLA-4, an immune checkpoint receptor, can bind to the B7 on the surface of APCs and then prevent the activation of CD4 T cells which deprives the costimulatory signal from CD28. Therefore, blockade of CTLA-4 can release T cells from suppression. So far, the predominant anti-CTLA4 antibodies involve ipilimumab and tremelimumab. According to a phase I/II clinical study (CheckMate-032), ipilimumab monotherapy presented a significant efficacy in patients with advanced GC with chemotherapy [36]. However, in another phase II clinical trial, maintenance therapy with ipilimumab presented no significant benefits for advanced GC [37]. Tremelimumab is another potential CTLA-4 inhibitor. According to a phase Ib/II trial, it has been reported that tremelimumab can promote the T-cell activity and showed a median PFS of 1.7 months and median OS of 7.7 months [38]. Although tremelimumab presents no such significant efficacy in GC patients, durable anti-tumor activity can be observed in several GC patients, which emphasized that GC patient with specific biomarker can obtain more benefits (Fig. 1).

### Dual ICI strategy

It has been revealed that the combination of anti-PD-1/PD-L1 and anti-CTLA-4 antibodies can promote an effective and durable response for a variety of cancers, especially in GCs. According to the previous study, the median duration of response in the combination of nivolumab plus ipilimumab outperformed than chemotherapy group in patients with *CPS* ≥ 5. In the CheckMate-032 study, the treatment of nivolumab was compared with the combination with ipilimumab in patients with advanced or metastatic tumors. Although the combination group presented higher ORR than the nivolumab alone, but the OS of these two groups was similar [10]. Notably, the benefit of combination therapy is more obvious in the patients with PD-L1-positive and microsatellite instability-high (MSI-H) features. Therefore, nivolumab combined with ipilimumab may be a potential treatment against GCs. However, there are also controversial results in CheckMate-649 trial. It has been revealed that combination of ipilimumab and nivolumab did not achieve the prolonged OS compared with the chemotherapy group [39, 40]. Moreover, according to the KEYNOTE-062 and KEYNOTE-061 trials, the combination of nivolumab and ipilimumab even increased the mortality rate in the early stage comparing to the chemotherapy group [19, 41]. Based on the above results, it has been revealed that combination of PD-1/PD-L1 and CTLA-4 monoclone antibody is not suitable for all cases with GCs. Therefore, different immunotherapeutic strategy may be adapted to specific populations (Table 1).

**Table 1:** ICIs monotherapy and dual strategies involving clinical trials against GCs

Trial	Patient feature	Agent	Target	Phase	Outcomes	Ref.
KEYNOTE-012 (NCT01848834)	Advanced GC	Pembrolizumab	PD-1	Ib	ORR (22%, 95% *CI* 10–39) and 13% with IRAEs	Muro et al. (2016) [34]
ATTRACTION-2 (NCT01928394)	Unresectable advanced or recurrent GCs	Nivolumab	PD-1	III	Positive for OS with 10% with IRAEs	Kang et al. (2017) [13]
KEYNOTE-059	Advanced GC after 2 or more lines therapies	Pembrolizumab	PD-1	II	Response rate 11.6% and complete response in 2.3%; 17.8% cases with IRAEs	Shitara et al. (2018) [42]
KEYNOTE-061 (NCT02370498)	Advanced GC; CPS ≥ 1	Pembrolizumab	PD-1	III	No significant improvement for OS than paclitaxel	Shitara et al. (2018) [11]
JAVELIN Gastric 100 (NCT02625610)	Advanced GC with 1st line maintenance	Avelumab	PD-1	III	No significant improvement for OS than chemotherapy	Moehler et al. (2021) [35]
NCT01585987	Untreated, unresectable, EGFR2-negative, locally advanced or metastatic GC	Avelumab	PD-1	III	No significant improvement for OS than chemotherapy	Moehler et al. (2016) [37]
POLARIS-02 (NCT02915432)	Advanced GC	Toripalimab	PD-1	Ib/II	Manageable safety profile and promising antitumor activity in advanced GC patients	Wang et al. (2019) [15]
CheckMate-577 (NCT02743494)	GC or gastroesophageal junction cancer (GEJC) after chemoradiotherapy	Nivolumab	PD-1	III	DFS was significantly longer in nivolumab group than placebo group	Kelly et al. (2020) [43]
CheckMate-032 (NCT01928394)	Unresectable advanced, recurrent, or metastatic GC or GEJC	Nivolumab, ipilimumab	PD-1; CTLA4	I/II	No significant improvement for OS than nivolumab alone	Janjigian et al. (2016) [44]
NCT02340975	Chemotherapy-refractory GC or GEJC	Durvalumab, tremelimumab	PD-1; CTLA4	Ib/II	Low response rates	Kelly et al. (2020) [38]

### Immunotherapy combined with other therapeutic strategy for GCs

#### Chemotherapy combination therapy

Currently, ICIs have been served as the neoadjuvant strategy before surgical resection or maintenance therapy after chemotherapy. According to the KEYNOTE-059 trial, the combination strategy presented a much higher ORR than pembrolizumab monotherapy. However, there was still a contrary in this trial. The pembrolizumab monotherapy appeared to have a longer OS than the combination group [45]. Based on the result of the phase III trial KEYNOTE-062, patients with untreated advanced or metastatic GCs were administrated with pembrolizumab and chemotherapy. However, the combination of pembrolizumab and chemotherapy failed to present superiority to chemotherapy alone in mOS in patients with PD-L1 *CPS* ≥ 1 or ≥ 10 [19].

The CheckMate-649 trial assesses the superiority of nivolumab plus ipilimumab or chemotherapy over chemotherapy alone in patients with HER2-negative cancers [39, 40]. According to the trial CheckMate-649, nivolumab plus chemotherapy presented superior OS compared to chemotherapy alone and reduce 20% of mortality. Moreover, in the patients with higher CPS, this combination strategy presented better efficacy with increased ORR and prolonged OS. Therefore, the nivolumab combination therapy has been approved by FDA for the patients with advanced or metastatic GC [46]. In addition, other anti-PD-1 antibodies also presented promising efficacy as the first-line treatment in patients with GCs. According to a phase 2 clinical trial, camrelizumab with CapeOx (oxaliplatin or capecitabine), followed by camrelizumab plus apatinib, presented an ORR of 65% in patients with advanced or metastatic GC [47]. So far, another multicenter phase III clinical study (NCT02942329) based on the camrelizumab plus apatinib as the second-line treatment against GCs is ongoing [48]. Moreover, in a phase Ib study, sintilimab with CapeOx was served as the first-line treatment and showed a great response against advanced or metastatic GC [49]. Another clinical trial revealed that combination of sintilimab with CapeOx as the neoadjuvant treatment achieved 23.1% pathological complete response (pCR) and 53.8% major pathologic response (MPR) in GC patients [50]. Therefore, further study is required to determine whether pCR can be a predictive factor for the long-term survival benefits. So far, an ongoing study (ORIENT-106) is underway to determine the efficacy of the combinative strategy of sintilimab and ramucirumab for progressive or metastatic GC (*CPS* ≥ 10). According to the NCT03469557 study, tislelizumab plus chemotherapy as the first-line treatment presented an ORR of 46.7% in GC patients [51]. According to the CS1001-101 study, it has been revealed that there was a potential correlation between the PD-L1 expression and the efficacy of immunotherapies. Moreover, the CS1001 (PD-L1 antibody) plus XELOX presented an ORR of 62% in patients with advanced GC [52]. Another ongoing study (NCT03852251) also reported that ICIs combined with mXELOX presented inspiring anti-tumoral efficacy in advanced GC patients [53]. In a summary, there is a great clinical value in the combination of immunotherapy with chemotherapy, and further clinical trials with large cohorts are also urgently required (Table 2).

**Table 2:** ICIs combined with traditional chemotherapies involving clinical trials against GCs

Trial	Patient feature	Agent	Target	Phase	Outcomes	Ref.
KEYNOTE-062 (NCT02494583)	Advanced GC/EGJC	Pembrolizumab + chemotherapy	PD-1	III	Pembrolizumab alone or plus chemotherapy was not superior to chemotherapy for the OS and PFS	Shitara et al. (2020) [19]
CheckMate-649 (NCT02872116)	Unresectable advanced GC	Nivolumab + chemotherapy	PD-1	III	Nivolumab plus chemotherapy presented superior OS compared to chemotherapy alone and reduce 20% of mortality	Moehler et al. (2020) [46]
NCT02937116	Locally advanced or metastatic GC	Sintilimab + CapeOx	PD-1	Ib	Sintilimab combined with CapeOx presented acceptable safety and promising efficacy	Jiang et al. (2020) [49]
NCT04065282	Advanced resectable GC/GEJ adenocarcinoma	Sintilimab + CapeOx	PD-1	II	Sintilimab plus oxaliplatin/capecitabine showed promising efficacy with encouraging pCR rate and good safety profile	Jiang et al. (2020) [50]
NCT03469557	Advanced GC	Toripalimab + CapeOx + tislelizumab + chemotherapy	PD-1	II	Tislelizumab plus chemotherapy presented durable responses with manageable tolerability	Xu et al. (2020) [51]
CS1001–101	Advanced GC/EGJC	CS1001 + XELOX	PD-1	I	CS1001 plus XELOX presented an ORR of 62% in patients with advanced GC	Shen et al. (2020) [52]
KEYNOTE-811 (NCT03615326)	Advanced GC/EGJC	Pembrolizumab with trastuzumab and chemotherapy	PD-1	III	Very preliminary data	Chung et al. (2021) [54]
NCT02954536	HER2-positive metastatic esophagogastric cancer.	Pembrolizumab with trastuzumab and chemotherapy	PD-1	II	pembrolizumab with trastuzumab and chemotherapy presented acceptable safety and promising efficacy against HER2+ cancer	Janjigian et al. (2020) [1]
UMIN-CTR	Advanced GC	Nivolumab with paclitaxel plus ramucirumab	PD-1	I/II	Nivolumab with paclitaxel plus ramucirumab presented promising antitumor activity with manageable toxicities	Nakajima et al. (2021) [55]

#### Targeted antibody combination therapy

Immunotherapy combined with targeted therapy is a heating topic in a variety of cancers. In the terms of GCs, HER2 and VEGF/VEGFR are the predominant optional targets in the clinical practice. Accumulative evidence has illustrated the synergistic effect of ICIs and anti-HER2 therapy in various cancers, including breast cancer and GCs. Anti-HER2 l antibody (trastuzumab) combined with chemotherapy is previously considered as the first-line option for advanced HER2-postive GCs. In the phase III clinical study KEYNOTE-811 (NCT03615326), pembrolizumab combined with trastuzumab, fluoropyrimidine, and platinum-containing chemotherapy presented a better ORR than the group administrated with trastuzumab combined with chemotherapy [56]. The efficacy of pembrolizumab combined with trastuzumab was evaluated for patients with HER2-positive advanced GC. Comparing with the trastuzumab and chemotherapy, combining with pembrolizumab achieved a significant tumor reduction. Based on the potentially clinical value of immunotherapy combined with targeted therapy, FDA approved that pembrolizumab plus trastuzumab and chemotherapy were served as the first-line treatment against HER2-positive GCs. In 2020, a phase II study PANTHERA is based on the first-line triple treatment regimen (pembrolizumab, trastuzumab, chemotherapy) in HER2-positive advanced GCs patients. According to the results, approximately 56.6% of patients presented more than 50% reduction in tumor burden [57]. Moreover, margetuximab is another optimized anti-HER2 antibody and mediates the activation of immune responses by anti-HER2 targeted T-cell responses. According to the phase II/III MAHOGANY trial, combination of margetuximab with the retifanlimab showed great anti-tumor effects [58].

VEGF/VEGFR inhibitors are another targeting option against GCs. So far, there are various VEGFR2 targeting drugs, including ramucirumab, apatinib, lenvatinib, and regorafenib [59, 60]. A phase I/II study based on the nivolumab combined with paclitaxel and ramucirumab revealed promising clinical efficacy. A multicenter phase I/II study of nivolumab combined with paclitaxel plus ramucirumab demonstrates promising clinical activity. Patients with higher PD-1 expression (*CPS* ≥ 1) presented longer OS [55]. According to the EGONIVO study, combination with regorafenib, nivolumab also achieved good response rate and OS in advanced GC patients [61]. Moreover, the combination of ramucirumab and durvalumab treatment has also been detected in GC patients. The results revealed that the safety of combinative strategy is consistent with the single treatment, which emphasized the clinical value of the combination of VEGF/VEGFR inhibitors with ICIs against GCs [62]. Except the anti-HER2 antibody and VEGF/VEGFR inhibitors, ICIs can also combine with other targets-based therapies. Moreover, when nivolumab combined with ramucirumab served as the second-line treatment for advanced GC, the results revealed that ORR was 26.7%, and OS was 9.0 months (Table 3).

**Table 3:** ICIs combined with target therapies involving clinical trials against GCs

Trial	Patient feature	Agent	Target	Phase	Outcomes	Ref.
NCT02942329	Advanced hepatocellular carcinoma (HCC), GC/EGJC	Camrelizumab + apatinib	PD-1, VEGF2	I	SHR-1210 and apatinib combination therapy present manageable toxicity	Xu et al. (2019) [48]
CP-MGAH22-05 (NCT02689284)	Locally advanced or metastatic, resectable, HER2+ GC	Margetuximab + pembrolizumab	PD-1, HER2	Ib/II	Synergistic antitumour activity with the combination of margetuximab with anti-pembrolizumab	Catenacci et al. (2019) [12]
EGONIVO (EPOC1603)	GC and colorectal cancer	Regorafenib + nivolumab	PD-1, VEGF2	Ib	Combination of regorafenib and nivolumab presented a manageable safety profile and encouraging antitumor activity	Fukuoka et al. (2019) [61]
EPOC1706	Metastatic or recurrent GC/EGJC	Lenvatinib + pembrolizumab	PD-1, VEGF2	II	Promising anti-tumor activity with an acceptable safety profile in patients with advanced GC	Kawazoe et al. (2020) [59]
LEAP-005 (NCT03797326	Advanced GC	Lenvatinib + pembrolizumab	PD-1, VEGF2	II	PFS with 2.5 months (1.8–4.2) and OS with 5.9 months	Villanueva et al. (2020) [60]
NCT02572687	Measurable GC	Ramucirumab + durvalumab	PD-1, VEGF2	Ia/b	Ramucirumab/durvalumab exhibited manageable safety and antitumor activity	Bang et al. (2020) [62]

#### Radiotherapy (RT) combination therapy

RT is a common therapeutic strategy to damage cancer cells directly and activate the immune responses. However, previous studies also revealed that RT can upregulate the expression of PD-L1, induce immunosuppression, and consequently counters the benefits from RT. Therefore, addition of ICIs in the process of RT presents the synergistic effect against cancer cells. According to the CheckMate-577 study, nivolumab as the adjuvant therapy combined with a triple regimen (neoadjuvant chemoradiotherapy sequential surgery) in GC patients, which showed great benefits in DFS [43, 63]. Another Neo-PLANET phase II clinical study applied the combination of SHR-1210 and chemoradiotherapy as the neoadjuvant treatment for locally advanced proximal GCs, and the pCR rate was 26.7%. Moreover, a series of clinical studies are undergoing to explore the efficacy of the combination of RT and immunotherapy.

### Toxicity profile and safety of ICIs in GC treatment

Accumulative evidence has revealed that ICIs are generally tolerated. Immunotherapy can provide lasting remission for patients with GCs; however, it sometimes brings life-threating adverse events, namely immunotherapy-related adverse events (IRAEs). The IRAEs are caused by the excessive inflammatory response and nonspecific reaction due to the ICIs. In the GCs, the IRAEs are consistent with the other cancers with immunotherapy. ICIs appears to present higher IRAEs after the combination with chemotherapies. Based on the Keynote-062 study, the combination group presents 24% of cases with IRAEs, but only 21% of cases can be observed in the pembrolizumab group. According to the clinical study, approximately 5–10% of IRAEs were induced by the anti-PD-1/PD-L1 antibodies against advanced GCs. Based on a phase III study, it has been reported that grade 3 or 4 IRAEs were increased up to about 10% in the immunochemotherapy group than the chemotherapy alone [64]. So far, the interstitial pneumonia and myocardial damage are attached much attention after the combination of anti-PD-1 antibodies and anti-HER2 therapy, but no other IRAEs are observed according to the KEYNOTE-811 trial [54, 65]. Of note, there are more IRAEs (about 35% of cases) which can be observed after the combination of anti-PD-1/PD-L1 antibodies and anti-CTLA-4 antibodies than single ICIs [66]. In addition, GM-CSF and IL-6 are demonstrated to be the potential targets to attenuate the toxicity and IRAEs from immunotherapy. Currently, it has been reported that the majority of IRAEs can be attenuated by the systemic corticosteroids and other ancillary strategies without impairing the clinical benefits of immunotherapy in GC treatment. Notably, there are certain relationship between the IRAEs and efficacy of ICIs. Therefore, further studies are required to determine such association [67].

## The other immunotherapeutic strategy

### Adoptive cell therapy (ACT)

Cancer cells can express several antigens with high immunogenicity, which leads to the activation of various immune responses. Therefore, cancer cells can be recognized and killed by the immune cells. However, current studies revealed that cancer cells can also release immunosuppressive factors, including lymphocyte-activation gene 3 (LAG-3), TGF-β, and IL-10, leading to the immune escape. Thus, as to the patients with low immune response towards cancer cells, adoptive cell therapy (ACT) may provide potential clinical value to treat GCs. The ACT utilizes a variety of immune cells, including cytokine-induced killer (CIK) cells and tumor-infiltrating lymphocytes (TILs) to effectively destroy cancer cells. CIK cells present with great anti-tumor activity and is responsible for the release of cytokines for the regulation of immune response. A previous clinical trial demonstrated the strong anti-tumor activity of CIK cells. Moreover, combination with targeted therapy may enhance the efficacy against the GCs. In addition, TILs have been widely applied in advanced gastric cancer. According to the previous study, combined therapies with tumor-associated lymphocytes can increase the survival rate to 50% than the traditional therapy [68, 69]. Moreover, currently, allogenic NK cells have also been established for the treatment of GCs [70]. However, this strategy is severely limited, due to the lack of strategies to obtain a large amounts of functional natural kill cells. Therefore, further studies are urgently required to establish novel approaches to obtain sufficient NK cells for cancer immunotherapy. Additionally, the immune cells, including expanded T cells and NK cells, or engineered chimeric antigen receptor T cells (CAR-T) and T-cell receptor T cells (TCR-T), can be utilized directly into the patients with cancers. Currently, CAR-T cells have been widely applied in clinical trials or experimental studies, because of its high specificity and great anti-tumor function. During this process, T cells will be engineered to target cancer cells. Notably, NK group 2 member D (NKG2D) ligand is widely localized in GC cell, which made it a specific target against GC. Therefore, the NKG2D-CAR-T cells can present great tumor killing function and can potentially be a novel therapeutic strategy against GCs [71]. HER2 is another critical target in patients with GCs. It has been reported that HER2-CAR-T cells also present great efficacy against GCs and can be an effective strategy towards the HER2-positive GCs [72]. So far, the majority of CAR-T therapies in solid tumors still remain in early stage; however, Claudin18.2 CAR-T therapy presented a breakthrough against cancers. Claudin18.2 is the specific membrane protein in the GC cells and can be served as the therapeutic target. According to a preclinical study, Claudin18.2 CAR-T therapy can remove the tumor in rodent models without toxicity [73]. Although the great efficacy of CAR-T cell therapy in hematological malignancies has been demonstrated, its clinical value in GC or the other solid tumors still need further investigation in the future [74, 75]. Moreover, some other GC-specific expression protein can also be the potential targets against GCs, including folate receptor 1 (FOLR1) and mesothelin (MSLN). Corresponding CAR-T therapies can also be established and investigated [76, 77]. TCR-T immunotherapy is a modified T-cell-based ACT. The TCR genes that can recognize the antigen of cancer cells were transduced into the T cells. However, due to the tumor heterogeneity, cancer cells present different antigens, which limited the wide application of TCR-T therapy. Although TCR-T therapy may own great advantages over CAR-T against solid tumors, so far, the majority of the TCR-T clinical trials are undergoing the phase I/II clinical trials.

### Cancer vaccines

Cancer vaccine is another novel active immunotherapy against the advanced GCs through activation of immune responses. The therapeutic cancer vaccines involve autologous tumor cell vaccines, dendritic cell (DC) vaccines, peptide vaccines, and genetically engineered vaccines. So far, the well-established cancer vaccines are mRNA vaccines, which can promote the expression of antigen and further induce immune responses. According to previous study, mRNA cancer vaccines companied with moderate adverse effects and great efficacy comparing to the chemotherapy or targeted therapy [78]. A previous study has reported the role of autologous tumor-derived Gp96 vaccination in patients with GCs. The results revealed that the vaccine group improved that DFS comparing to the chemotherapy group [79]. Moreover, the combination of cancer vaccines with chemotherapies also presents significantly enhanced cytotoxicity against cancer cells [80].

### Challenges and potential strategies in immunotherapy against GCs

Accumulative clinical trials and experimental studies have revealed the great advantages of immunotherapies than the traditional therapeutic strategies against the GCs. However, there is no denying that there still exists a variety of challenges which limited the clinical application of immunotherapy, especially in GCs. The autoimmune toxicity and adverse effects of ICIs and CAR-T therapies required further attention, despite the synergistic effects on advanced GCs of combination treatment with ICIs and targeted therapy. However, VEGF, for example, presents with a wide expression, and the specific inhibition may commonly lead to the adverse effects, including hypothyroidism, coagulation disorders, hypertension, and neurotoxicity [81]. Moreover, as to the cancer vaccine, although favorable benefits have been reported in phase I/II trials against advanced GCs, the host immune response also limited its clinical efficacy. Therefore, novel strategies are required to overcome this limitation. The combination of cancer vaccine with other immunomodulators may prevent the immune suppression or with the chemotherapy to enhance anti-tumor effects and reduce cytotoxicity [82]. Of note, the CAR-T therapy presents amazing efficacy against GCs but also companied with strong toxicity [83]. Aiming to facilitate the clinical application of this effective immunotherapy, reduction of the toxicity from CAR-T therapy with shorter lifespan or “on-switch” is necessary [84].

## Conclusions

With the development of ICIs and the other immunotherapeutic strategies, there is an obvious change in the therapeutic options and efficacy against GCs. Although the ICIs, like anti-PD-1/PDL1 therapy, are not likely to be first-line treatment, they present great potential and clinical value in the combination with other therapies for the patients with advanced GCs. So far, the other immunotherapeutic strategies are not as mature as ICIs, but with the further development and investigation, better combination with ICIs may obtain better clinical outcomes against solid tumors. In a conclusion, based on the positive results in various clinical trials, immunotherapy has been incorporated in the clinical management of advanced GC. Further studies are urgently required to optimize immunotherapy efficacy, overcome emerging PD-1/PD-L1 resistance, and further promote GC patients’ outcomes.

## Data Availability

Not applicable. Data sharing is not applicable to this article as no datasets were generated or analyzed during the current study.

## Abbreviations

GC: Gastric cancer
ICIs: Immune checkpoint inhibitors
PD-1: Programmed cell death protein-1
CTLA-4: Cytotoxic T-lymphocyte-associated antigen 4
TMB: Tumor mutational burden
EBV: Epstein-Barr virus
TPS: Tumor proportion score
OS: Overall survival
MSI-H: Microsatellite instability-high
FOXP1: Forkhead box protein P1
HER2: Human epidermal growth factor receptor 2
TAMs: Tumor-associated macrophages
DCs: Dendritic cells
TCGA: The Cancer Genome Atlas
MPR: Major pathologic response
